# The reliability of the pass/fail decision for assessments comprised of multiple components

**DOI:** 10.3205/zma000984

**Published:** 2015-10-15

**Authors:** Andreas Möltner, Sevgi Tımbıl, Jana Jünger

**Affiliations:** 1 Ruprecht-Karls-Universität Heidelberg, Kompetenzzentrum Prüfungen in der Medizin Baden-Württemberg, Heidelberg, Deutschland

**Keywords:** Assessments, Decision accuracy, Decision consistency, Pass-fail reliability

## Abstract

**Objective: **The decision having the most serious consequences for a student taking an assessment is the one to pass or fail that student. For this reason, the reliability of the pass/fail decision must be determined for high quality assessments, just as the measurement reliability of the point values.

Assessments in a particular subject (graded course credit) are often composed of multiple components that must be passed independently of each other. When “conjunctively” combining separate pass/fail decisions, as with other complex decision rules for passing, adequate methods of analysis are necessary for estimating the accuracy and consistency of these classifications. To date, very few papers have addressed this issue; a generally applicable procedure was published by Douglas and Mislevy in 2010.

Using the example of an assessment comprised of several parts that must be passed separately, this study analyzes the reliability underlying the decision to pass or fail students and discusses the impact of an improved method for identifying those who do not fulfill the minimum requirements.

**Method: **The accuracy and consistency of the decision to pass or fail an examinee in the subject cluster Internal Medicine/General Medicine/Clinical Chemistry at the University of Heidelberg’s Faculty of Medicine was investigated. This cluster requires students to separately pass three components (two written exams and an OSCE), whereby students may reattempt to pass each component twice. Our analysis was carried out using the method described by Douglas and Mislevy.

**Results: **Frequently, when complex logical connections exist between the individual pass/fail decisions in the case of low failure rates, only a very low reliability for the overall decision to grant graded course credit can be achieved, even if high reliabilities exist for the various components. For the example analyzed here, the classification accuracy and consistency when conjunctively combining the three individual parts is relatively low with κ=0.49 or κ=0.47, despite the good reliability of over 0.75 for each of the three components. The option to repeat each component twice leads to a situation in which only about half of the candidates who do not satisfy the minimum requirements would fail the overall assessment, while the other half is able to continue their studies despite having deficient knowledge and skills.

**Conclusion:** The method put forth by Douglas and Mislevy allows the analysis of the decision accuracy and consistency for complex combinations of scores from different components. Even in the case of highly reliable components, it is not necessarily so that a reliable pass/fail decision has been reached – for instance in the case of low failure rates. Assessments must be administered with the explicit goal of identifying examinees that do not fulfill the minimum requirements.

## Zusammenfassung

**Zielsetzung: **Die gravierendsten Konsequenzen für einen Studierenden bei einer Prüfung besitzt die Entscheidung über „bestanden“ oder „durchgefallen“. Wie die Messzuverlässigkeit der Punktwerte muss bei qualitativ hochwertigen Prüfungen deshalb auch die Zuverlässigkeit der Entscheidung „bestanden“ oder „durchgefallen“ bestimmt werden.

Oft setzen sich Prüfungen eines Fachs (Leistungsnachweise) aus mehreren Teilprüfungen zusammen, die z. B. unabhängig voneinander bestanden werden müssen. In diesem Fall einer „konjunktiven“ Verknüpfung der Einzelentscheidungen „bestanden/durchgefallen“ wie auch bei anderen komplexen Bestehensregelungen sind zur Abschätzung der Genauigkeit und Konsistenz der Entscheidung „bestanden/durchgefallen“ adäquate Auswertungsverfahren erforderlich. Bislang liegen zu dieser Problemstellung nur wenige Arbeiten vor, ein allgemein verwendbares Verfahren wurde 2010 von Douglas und Mislevy publiziert.

In der Studie soll am exemplarischen Beispiel einer zusammengesetzten Prüfung, bei der mehrere Teilprüfungen unabhängig voneinander bestanden werden müssen, eine Analyse der Zuverlässigkeit der Entscheidung „bestanden/durchgefallen“ durchgeführt und Konsequenzen für eine verbesserte Methodik zur Identifikation von Studierenden, die die gestellten Mindestanforderungen nicht erfüllen, diskutiert werden.

**Methodik: **Untersucht wird die Entscheidungsgenauigkeit und -konsistenz von „bestanden/durchgefallen“ des Leistungsnachweises Innere Medizin/Allgemeinmedizin/Klinische Chemie der medizinischen Fakultät Heidelberg. Für diesen müssen drei Teilprüfungen (zwei Klausuren und ein OSCE) unabhängig voneinander bestanden werden, wobei jede Teilprüfung für sich zweimal wiederholt werden kann. Die Analyse erfolgt mit dem Verfahren von Douglas und Mislevy.

**Ergebnisse: **Auch bei hohen Reliabilitäten von Teilprüfungen lässt sich bei komplexen logischen Verknüpfungen der Einzelentscheidungen „bestanden/durchgefallen“ im Fall niedriger Nichtbestehensquoten häufig nur eine geringe Zuverlässigkeit der Gesamtentscheidung erreichen. So ist im hier untersuchten Beispiel trotz der bei allen drei Teilprüfungen guten Reliabilitäten von über 0,75 die Entscheidungsgenauigkeit und -konsistenz bei konjunktiver Verknüpfung der drei Prüfungsteile mit κ=0,49 bzw. κ=0,47 relativ niedrig. Die Möglichkeit, die Teilprüfungen jeweils zweimal zu wiederholen, führt dazu, dass von den Studierenden, die den Mindestanforderungen nicht genügen, nur etwa die Hälfte endgültig die Gesamtprüfung nicht bestehen würde, die andere Hälfte jedoch trotz mangelhafter Kenntnisse/Fertigkeiten ihr Studium fortsetzen kann.

**Schlussfolgerung: **Das Verfahren von Douglas und Mislevy erlaubt, Entscheidungsgenauigkeit und -konsistenz komplexer Verknüpfungen von Teilprüfungen zu analysieren. Auch bei hochreliablen Teilprüfungen wird – etwa im Fall niedriger Nichtbestehensquoten – nicht notwendigerweise eine zuverlässige Entscheidung über „bestanden“ oder „durchgefallen“ erreicht. Hierzu müssten Prüfungen mit dem expliziten Ziel der Identifizierung von Studierenden, die den Mindestanforderungen nicht genügen, durchgeführt werden.

## 1. Introduction

Assessments are performance measurements and possess, like all measuring instruments, only a limited accuracy. This must be sufficiently high so that the scores given for assessments reflect the content. Established methods exist for estimating the measurement reliability of the points given on assessments (e.g. Cronbach’s α); however, the reliability of the pass/fail decision is hardly taken into consideration in the analysis or evaluation of assessments.

This is remarkable insofar as precisely this aspect clearly has more importance for students in regard to their studies than the measurement reliability of a point value; failing an exam leads to remedial work, lost time, and under circumstances the question of whether to continue or quit medical school. This decision also has importance for the institution administering the assessment: if the examinee possesses the required knowledge and skills to continue the study program, an unjustified failure leads to a greater amount of work. If an examinee is allowed to pass despite not having the qualifications, then not only significant problems in continuing university study are to be expected, but even the endangerment of medical patients in worst case scenarios (see [5]).

Presumably, this topic has also received so little attention in Germany in relation to medical education because the regulations of the medical licensing act (*Ärztliche Approbationsordnung*) have been generally adopted by the academic rules and regulations of most medical schools for multiple-choice testing, the longstanding dominant testing format. With the purely formal definition of a passing score being 60% of all questions asked, this approach does not permit a content-based, criterion-oriented definition of the minimum requirements. To our knowledge, in Germany only the rules and regulations of the Medical Faculty at the University of Heidelberg allow standard setting for multiple-choice tests; this means the ability to deviate from the formal rule of 60% to pass and define a passing score according to content-based criteria and in a standard procedure, similar to the established standard setting for an OSCE [2], [5].

The establishment of new testing formats, with which practical skills, qualifications and necessary competencies for practicing medicine should be assessed in addition to pure subject knowledge, demands definition and, for assessments, the practical setting of minimum requirements. As a result, it is also necessary to pay close attention to the decision accuracy, decision consistency and pass-fail reliability when evaluating tests or testing formats [19]. The decision accuracy indicates the extent to which the examinees that satisfy the minimum requirements pass an actual test and the examinees without sufficient knowledge fail. Decision consistency refers to the agreement of pass/fail between two equivalent tests, meaning two tests that measure *the same knowledge or the same skills equally well*. It needs to be noted here that “same” does not imply that the tests only cover one construct in terms of test theory. An OSCE can contain stations dealing with practical skills and with communicative competencies which are to be regarded as subscales in terms of test statistics. An equivalent test must then have practical and communication stations with the same scope and of the same difficulty.

A series of methods has been developed, particularly since the 1980’s, to ascertain the accuracy and consistency in respect to individual tests, even though none of these methods can be viewed as the standard procedure (see [6], [13], [14], [16], [18], [23], [25]). To obtain graded course credit in many medical subjects, multiple individual assessments must be taken, for instance a written exam covering theoretical knowledge and an OSCE to assess practical skills. If these assessment results are combined together into an overall score through weighted averaging or totaling, the entire assessment can be treated as one “single” test.

Often there is another approach, completely justified in terms of content: instead of *compensatory* combination of assessment scores, *all the individual assessments must be passed*. This *conjunctive combination* (logical “and” conjunctions) of the pass/fail decisions has significant effects on the accuracy/consistency of the overall decision, since one single unreliable decision on an individual test can ruin the reliability of the overall decision:

*...because longer collections of test questions tend to be more reliable than shorter collections of test questions, compensatory scoring tends to be more reliable than conjunctive scoring. In conjunctive scoring, if a student has to pass all of the content areas separately, the least reliable score controls whether a student will pass.* [26]

Practical instances of this include subjects that spread the tested content out over multiple tests to limit the scope of a particular test and subjects in which both theoretical knowledge and practical skills are imparted resulting in a written assessment for the theory and a practical one for the skills. Instead of allowing compensatory scoring in these cases, requiring students to satisfy the minimum on each separate assessment is often justified. Ultimately, a conjunctive combination will also be used for the entire course of study: only those who *have passed in all of the subjects*, will successfully complete the degree program.

Assessment scores can also be combined in other ways. Alongside the conjunctive combinations already mentioned, disjunctive (logical “or” conjunctions) are also possible when only one single component of many must be passed. An example of this would be the repeated assessments. If an assessment can be retaken once, a student has passed if it is passed on the first or second attempt (that a student need not appear for the second administration if he or she has already passed the first attempt is of no interest to logic). In practice at schools and universities even more complex rules apply, such as graded credit must be successfully attained for three of five possible courses.

Only a few studies exist regarding the decision reliability for complex combinations of assessment scores [24], a generally applicable method of analysis has been proposed by Douglas and Mislevy [7], [8]. Our study applies this method to analyze the assessment for the subject cluster General Medicine/Internal Medicine/Clinical Chemistry that was given at the Faculty of Medicine in Heidelberg during the winter semester 2012-13 and, for the attainment of which, two written exams and one OSCE had to be passed separately. Students had the option of repeating each individual component of the assessment twice.

Graded credit for a cluster of subjects (*fächerübergreifende Leistungsnachweis* or FÜL) is particular to the German medical licensing regulations (*Approbationsordnung*), according to which every medical school must bundle multiple course subjects into one instance of graded course credit. This legal requirement is without significance for the following statistical observations. Douglas and Mislevy’s method is directed toward the accuracy and reliability of a complex pass/fail decision that is the result of a combination of individual decisions. Regardless of the formal legal definitions of a FÜL, the terms “overall test” (for full graded credit) and “individual test” or “component” (for the individual subject assessments) will be used.

The aim of this study is to present a suitable method for the analysis of pass/fail decision reliability using the example of a bundled assessment and establish it as an essential aspect of ensuring the quality of tests.

## 2. Principles

### Decision accuracy and decision consistency

Our starting point is the assumption that the examinees can be classified according to their knowledge or skills into two subgroups, one that fulfills the minimum requirements (master, competent examinee) and one that does not fulfill them (non-master, incompetent examinee). For an assessment in a particular subject, such a definition could be taken from a catalogue of learning objectives, with the definition of a master being someone who – for example – masters 70% of these learning objectives.

For an actual assessment, learning objectives are selected for testing and a passing score is defined. The lowest passing score could then also be set at 70%. A student who has mastered 90% of all learning objectives would with great probability exceed this cut-off, in contrast to someone who has mastered 72% – thus also fulfilling the minimum requirements (master) – but who could possibly be unlucky and fail. The same applies to students who are just under the cut-off for master status, but pass with a bit of luck. A more detailed discussion of the difference between the definition of master (performance standard) and the passing score can be found in [12] (see also [2], [5]).

Depending on the objective of the assessment, the passing score can be varied. If a higher passing score is set, the probability of a non-master passing is reduced, but at the same time the risk of inaccurately classifying a master as a non-master increases. This is analogous to a diagnostic test that compares a gold standard (in this situation the knowledge that a person is a master or non-master) with an actual test score. If one regards the assessment as the diagnosis of non-masters, then this test possesses a certain sensitivity (the probability of failing non-masters) and a specificity (probability that a master passes). Changes to the cut-off point for the test value lead to an increase or decrease in the sensitivity, along with a simultaneous decrease or increase in the specificity.

The degree to which masters and non-masters can be identified using the assessment is referred to as the decision accuracy. The left contingency table in Table 1 (Tab. 1) presents in full the relative proportions for master/test passed, master/test failed, non-master/ test passed, and non-master/test failed.

If two *equivalent* tests are administered, then the degree of agreement between the two test scores is the *decision consistency* or pass-fail reliability. The corresponding contingency table is shown on the right in Table 1 (Tab. 1). If the tests are equivalent, then the proportion of students who pass the first test and fail the second must be exactly the same size as the proportion that failed the first and passed the second.

The two values most frequently used in the literature for decision accuracy and decision consistency are the relative number of correct decisions *P*_a_ (corresponding to the correct classification rate for diagnostic tests) and agreements *P*_c_ [11] and Cohen’s κ [4] (for its use in connection with the sensitivity and specificity of diagnostic tests, see [3]). Cohen’s κ corrects the number of correct decisions P_a_ and the agreements P_c_ for the effects of chance that can be expected in the margin totals of the contingency table. The corresponding values are designated by κ_a_ and κ_c_.

κ assumes the value of 1 in the case of complete agreement. The application of κ as a measure of agreement is criticized in some places (e.g. [10]) and alternatives have been propagated. In our opinion, all the coefficients in this context come with the disadvantage that, with reduction to a single index, important information is lost. Therefore, when analyzing a test, the *entire contingency table* should be drawn upon.

#### Procedures for estimating the decision accuracy and consistency for individual assessments

In the literature, many methods are presented for determining decision consistency for individual tests. Known are those presented by Livingston-Lewis [16] and Peng-Subkoviak [18]. Overviews and comparisons also exist [6], [13], [14], [23], [25]. In our opinion, it is not currently possible to show a clear preference for any particular one among the various methods.

#### The method of Douglas und Mislevy

Douglas und Mislevy’s method [7]‚ [8] serves to determine the decision accuracy and consistency for complex decision rules based on scores from multiple tests. The pre-requisite is that the data of the individual tests can be described by a multivariate normal distribution and the reliabilities of the tests are known. In practice, however, scores are not normally distributed, which is why an adequate transformation of the data must be undertaken. For a precise description of the method, reference must be made to the original literature [7], [8].

**Figure 1:**
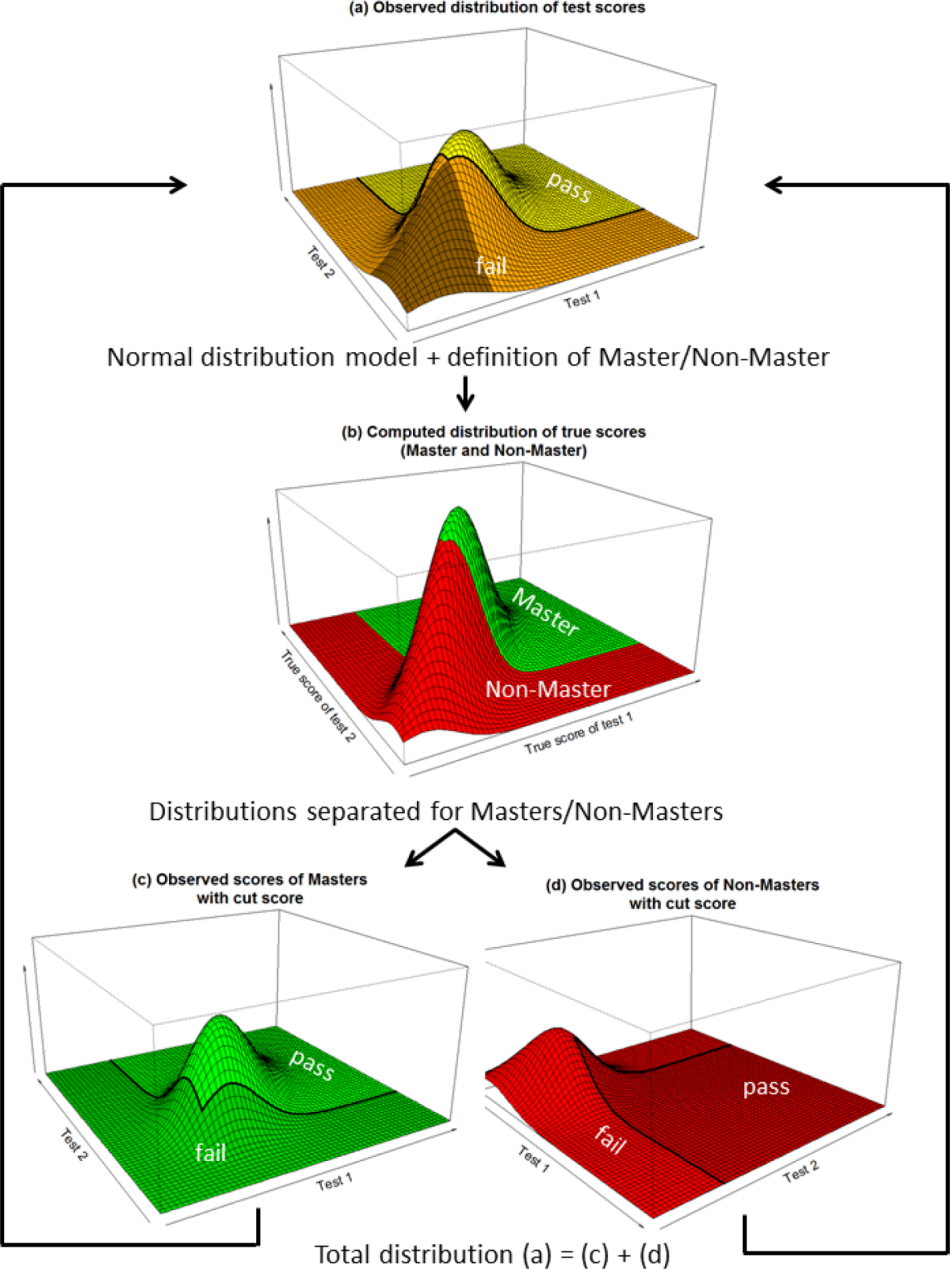
The steps for the method of Douglas and Mislevy: (a) distribution of the test scores for two tests; (b) estimation of the true values and definition of master/non-master according to the model; (c) distribution of the scores achieved by masters; (d) distribution of the scores achieved by non-masters (note: perspective is different). The distribution of the overall results (a) is comprised of the scores achieved by masters and non-masters.

For the purpose of understanding, let us take a simple, fictional example to determine the decision accuracy with graphic illustration of two individual tests (see Figure 1 (Fig. 1)). Those who passed both individual tests have passed overall (conjunctive combination).

Figure 1a (Fig. 1) illustrates the distribution of the scores. The examinees whose scores lie within the yellow part of the curve have passed both individual tests and have thus passed overall (in Table 1 (Tab. 1) this is represented by a_1+2_). Orange denotes the area of the distribution in which one individual test was passed and one was not. These examinees have not passed overall, just as those who did not pass either of the individual tests (brown area). The proportion of those in the L-shaped section of the curve (orange and brown) – representing those who failed overall – is represented by a_3+4_ in Table 1 (Tab. 1).

In the method proposed by Douglas and Mislevy, the distribution of the “true values” is determined according to the model of classical test theory and the assumption of normal distribution, meaning the distribution of the values if these had been measured without any error. For this, the reliabilities of the individual tests must be known. The resulting distribution shows a distinctly lower variance. The masters and non-masters are defined on the level of the true values. Figure 1b (Fig. 1) shows this distribution: the masters are those who satisfy the minimum requirements for both tests (green area; a_1+3_ in Table 1 (Tab. 1)), while non-masters are those who have not satisfied the minimum requirement for one area of both individual tests (red, L-shaped area; a_2+4_ in Table 1 (Tab. 1)).

To determine the decision accuracy, the model now examines how the masters’ scores are distributed (see Figure 1c (Fig. 1)). Due to errors of measurement in the tests, a portion of the masters failed (dark green area). The light green area shows the group of masters who passed overall (a_1_ in Table 1 (Tab. 1)); the dark green area indicates the masters who failed overall (a_3_ in Table 1 (Tab. 1)).

The corresponding graph for the non-masters is presented in Figure 1d (Fig. 1). This is presented from another perspective to make the borderlines more visible. The light red area indicates the portion of non-masters who did not pass overall and dark red those who did pass overall (a_4_ and a_2_ in Table 1 (Tab. 1)).

If one combines the distributions of the masters and non-masters in Figures 1c and 1d, then the overall distribution of the test scores in Figure 1a (Fig. 1) is seen again.

## 3. Method

### 3.1 Data

The aim of this study is to analyze the scores given for the graded course credit in the bundled subjects of Internal Medicine/General Medicine/Clinical Chemistry at Heidelberg University’s Faculty of Medicine during the winter semester 2012-13. The graded assessment consists of the written exam in Internal Medicine/General Medicine, an oral practical assessment (OSCE), and the written exam in Clinical Chemistry. To receive the graded course credit, a case report, a MiniCEX, and Encounter Cards to assess professionalism are also required. Since the pass rate is 100% for each of these, they are of no relevance to this investigation. Only the students who took all three tests were included in the analysis (*N*=147). The basic data for the tests are presented in Table 2 (Tab. 2). All in all, seven of the 147 examinees who sat for all three tests failed at least one of the components.

For the written exams in the subjects Clinical Chemistry and Internal Medicine, masters were defined as those who would correctly solve 60% of the questions from the particular question pool for each subject. In terms of the OSCE, the definition of master was those whose mean point totals for the OSCE stations in the subject was at least the number of points set as the standard (performance standard, [5]).

The passing scores for each written exam were defined as 60% of the possible points for the questions actually posed; for the OSCE, the passing score was the mean of the number of points defined as the standard for the stations used (passing score).

#### 3.2 Statistical analysis

The analysis of the accuracy and consistency of the pass/fail decision was mainly carried out according to the method proposed by Douglas und Mislevy [7], [8].

The method applied by Douglas and Mislevy makes no assumptions about the internal structure of the individual tests in terms of test theory, or about that among the individual tests. In particular, the individual tests are neither required to be homogenous or one-dimensional, nor must a uniform performance dimension be represented by the entirety of the components. However, it is pre-requisite that the data is sufficiently well described by a normal curve of distribution and the measurement reliabilities (reliabilities) of the individual tests are adequately estimated.

Since the point values of the tests each deviate in a highly significant manner from normal distributions (Shapiro-Wilk tests: all *p*<0.0008), the data were subjected to a multivariate Box-Cox transformation [1]. For the transformed data, a test for deviation from trivariate normal distribution using the generalized Shapiro-Wilk test as described by Villasenor-Alva and Gonzalez-Estrada [22] revealed a *p*-value of 0.8467 (MVW=0.9929), so that a sufficiently good adjustment of the data can be assumed. In contrast to the normalizing rank transformations applied in the study by Douglas and Mislevy, an adjustment to a *multivariate* normal distribution is aimed for with this transformation. To estimate the reliability of the individual tests, Guttman’s λ_2_ was selected as the coefficient allowing for a slightly better estimation of the minimum reliability than Cronbach’s α (=Guttman’s λ_3_) [9].

The contingency tables for the decision accuracy and consistency of the *individual tests* and their *conjunctive combination* were calculated using numerical integration of the multivariate normal distributions with the algorithm of Miwa, Hayter and Kuriki [17].

*Taking the two options* to repeat each individual test into account, this analysis is only of a theoretical nature insofar as it is assumed that students, who have not passed a test, concentrate on learning for the repeat attempt. In the analysis undertaken here it is assumed that the students taking these tests sit for the second attempt with the same knowledge they possessed for the first. The algorithm of Miwa et al. [17] is unsuited for the integration of a higher-dimensional normal distribution necessary for calculating the statistical values, so this analysis was done with Monte-Carlo integration as in [8]. All in all, 100,000 simulated data sets were generated to ensure sufficient accuracy of the results.

## 4. Results

### 4.1 Individual tests

The contingency tables in Table 3 (Tab. 3), Table 4 (Tab. 4) and Table 5 (Tab. 5) cover the individual tests. The estimated number of failing examinees resulting from the model of normal distribution is calculated as the failure rate of the model ×N=0.0331×147=4.9 for the written exam in Internal Medicine, 3.0 for Clinical Chemistry, and 1.9 for the OSCE. It can be seen that these rates deviate only slightly from the number of examinees who actually failed: 4, 5 and 1 (see Table 2 (Tab. 2)). For all three tests, Cohen’s κ coefficients κ_a_ (decision accuracies) and κ_c_ (decision consistencies) are low.

#### 4.2 Assessments composed of multiple scores

##### 4.2.1 Conjunctive combination of the individual tests

The decision accuracy and consistency for the conjunctive combination of the three tests are presented in Table 6 (Tab. 6). According to the model of Douglas and Mislevy, it is to be expected that 7.8 examinees would fail (=failure rate of the model ×N=0.0531×47=7.8). Seven candidates did indeed fail (many of the students did not pass more than one test), demonstrating satisfactory agreement between the model and the actual data. The test logic leads to a clear classification of the students who do not meet the requirements; the proportion of non-masters who pass all three tests is now 0.004 in total (although consideration must be given to the fact that their overall proportion is only 0.0232). The sensitivity to uncover non-masters is 82%, the specificity 97%; however, the positive predictive value is low with 36%.

The decision consistency (three administrations of equivalent tests) does not reach a satisfactory value with κ_c_=0.474. Classification of 94.7% of the examinees would occur right off (*P*_c_), meaning that conflicting information would exist for 5.3% of the examinees about successfully achieving the full graded credit.

##### 4.2.2 Complex conjunctive and disjunctive combination for repeat tests

**Figure 2:**
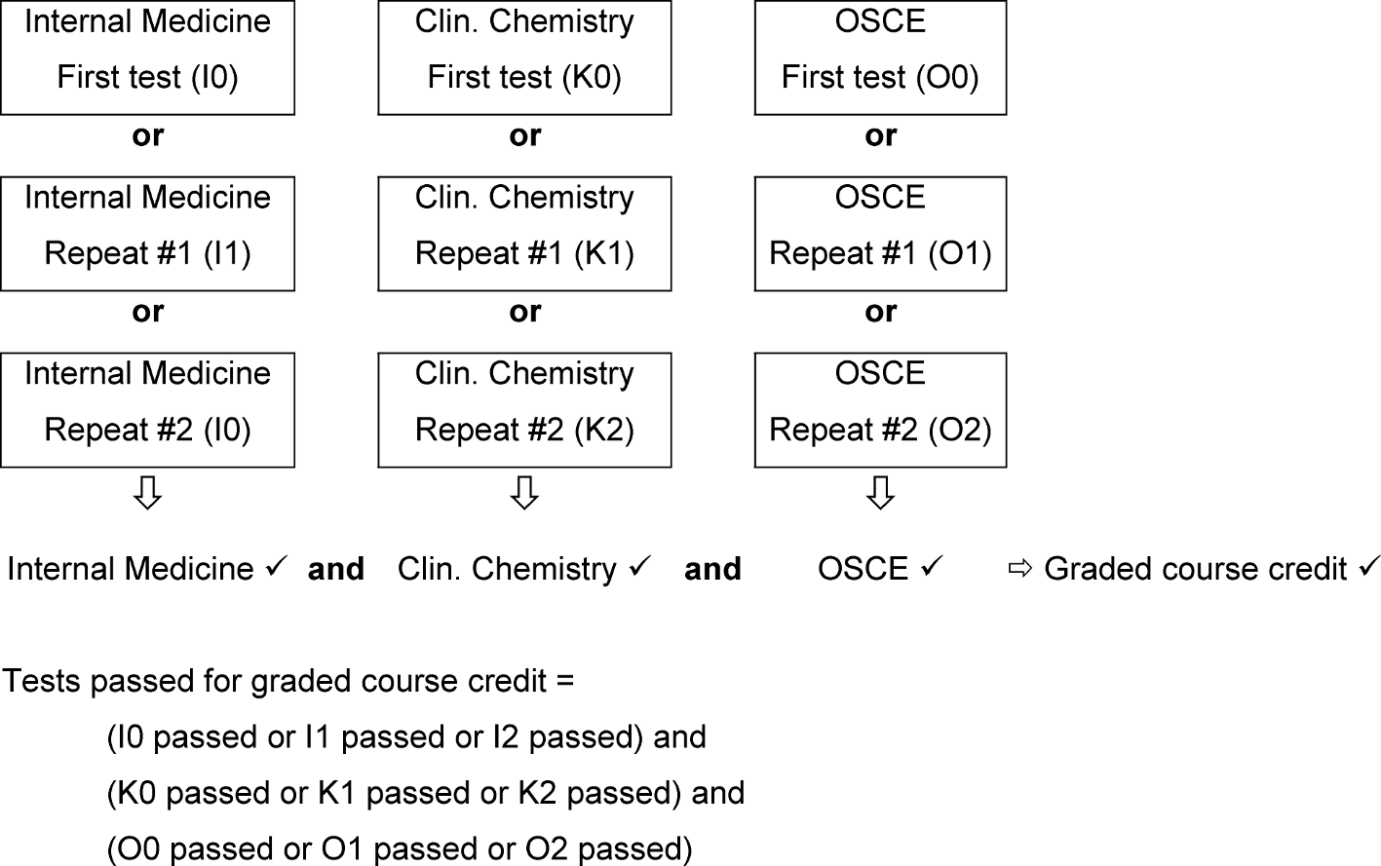
Decision rules for obtaining graded course credit for the subject cluster Internal Medicine/General Medicine/Clinical Chemistry.

Each of the three tests in Internal Medicine, Clinical Chemistry and the OSCE can be retaken a total of two times before the student has definitively failed. Logically, this means that a student must pass one of three written exams in Internal Medicine, one of three written exams in Clinical Chemistry, and one of three OSCEs. Within each testing format, the pass/fail decision is then disjunctively combined, and the three component decisions thus conjunctively (see Figure 2 (Fig. 2)). The fact that a student who has passed a test on the first attempt does not appear for another attempt in the same subject is not of importance to the decision logic.

Table 7 (Tab. 7) contains the contingency tables for the decision accuracy and consistency with the assumption that a student takes all tests with the same level of knowledge.

Of significance here is primarily that of the 2.32% of the students (a_2+4_=0.0232) who do not meet the requirements (non-master) more than half (a_2_=0.0124) would ultimately receive the graded credit, meaning that, as a result of the possibility to repeat tests, only a portion of the students who do not fulfill the requirements are stopped from continuing the program (note the substantial difference in regard to the results in the section above, in which the corresponding value with a_2_=0.0040 in Table 6 (Tab. 6) is clearly lower than the value of 0.0124 in Table 7 (Tab. 7)).

## 5. Discussion

### Individual tests

*Decision accuracy:* all three of the individual tests demonstrate an overall satisfactory reliability (see Table 2 (Tab. 2)). Of the non-masters, who altogether represent only 0.5 – 1.8% of the examinees (see Tables 3 (Tab. 3) to 5: a_2+4_), about one-third pass each of the tests (a_2_). The relevant percent of the masters who do not pass the test is low in all cases (a_3_); however, in absolute numbers this is distinctly more than there are non-masters taking the test, so that for all three tests more than double the number of assumed non-masters in the group fail.

*Decision consistency: *The reliability of the decision to fail an examinee must be assessed as unsatisfactory. Of those who fail, about 60–65% would pass an equivalent repeat test. The poor decision consistency is also seen in the low κ_c_ values of 0.33–0.41.

#### Conjunctive and complex combinations of the test scores

*Decision accuracy: *The contingency tables regarding the decision accuracy for the conjunctive combination of the three tests (see Table 6 (Tab. 6)) show that of the 2.3% non-masters (the students who do not meet the minimum requirements in at least one of the three subjects) only 17% pass (a_2_/a_2+4_=0.040/0.232=0.0172). The relative percent of masters who fail though increases to 3.5% (a_3_/a_1+3_=0.0348); for the individual tests this percent was at the highest 2%. In this case also, distinctly more examinees fail (5.3%, a_3+4_) than there are non-masters among the candidates. Cohen’s κ_a_ is with 0.49 almost just as high as the value of the best κ_a_ for the individual tests (written exam in Internal Medicine); the percentage of correct classifications is lower with *P*_a_=0.96. According to this, the assertion that in conjunctive combinations the test with the poorest decision accuracy dominates must be evaluated with more precision.

If the fact that each student has two opportunities to repeat a test is taken into consideration (see Table 7 (Tab. 7)), then assuming that the students attend equivalent repeat tests with the same level of knowledge or skills, only 47% of the non-masters do not in the end receive the graded credit (a_4_/a_2+4_=0.0108/0.0232=0.4655). For the masters, this is negligibly small with 2.3‰ (a_3_/a_1+3_=0.0022/0.9768=0.0023). Thus, the testing structure with the two options to retake each individual test is obviously poorly suited for reliably recognizing the non-masters.

*Decision consistency: *when conjunctively combining the three tests, the stability of the decision “fail” is also not satisfactory, but somewhat better than for the individual tests. In the case of an equivalent test complex consisting of the tests in the three subjects, a little more than half the examinees would pass the test. If κ_c_ is used as the consistency index, then this is higher than for each individual test with a value of 0.47.

When taking the possibility to repeat tests into account, a similar situation emerges: only somewhat more than half of the students who ultimately fail would be forced to end their studies again if they started over from the beginning.

#### Summary

In conclusion, it is clear that the pass/fail decision for the tests administered here needs improvement not only in terms of its accuracy, but also its consistency. “Sifting out” the non-masters is not possible in a reliable manner because tests may be repeated. On the other hand, there is hardly any danger that someone who meets the requirements will have to discontinue university study due to one or more instances of bad luck on tests.

To start with, the reason for this result could be seen in the model of normal distribution. To achieve an acceptable decision accuracy and consistency in the case of low failure rates, an extremely high reliability is necessary (a corresponding table for the κ_c_ coefficients is presented in [21]). This characteristic however is not specific for the normal distribution; not presented here are analyses for other assumed distributions that lead to similar results. Making the usual assumptions about the distribution form of the point totals on tests, most non-masters will fall close to the passing score if there is a *low failure rate and no excessively high reliabilities*. This does not depend on whether a formal (e.g. required by law), norm-oriented, or criterion-oriented cut-off is involved. This is why there is a relatively high probability that non-masters pass with a bit of luck, so that high levels of accuracy or consistency cannot be expected in these cases.

#### Limitations of Douglas und Mislevy’s method

The major limitation of the method proposed by Douglas und Mislevy is its assumption of a multivariate normal distribution. For the tests analyzed here, an acceptable normalization of the data was possible through a multivariate Box-Cox transformation, something that would not work in every case for data from other tests. Furthermore, the assumption of a multivariate normal distribution for the true values and measurement errors implies a constant error of measurement. However, the error of measurement can be distinctly higher at the cut-off point and lead to an overestimation of the decision accuracy. On the other hand, the distributions of the observed point values are clearly skewed to the left. As a result of the normalizing transformation, the values for very poor students have been moved “closer” to the passing score, so that in the analysis they belong more to the group for which, due to the error of measurement, inaccurate or inconsistent decisions are to be expected, although on the original scale they are reliably identified as non-masters.

#### Low decision accuracy and consistency: consequences for testing

With low failure rates, as for the tests analyzed here, a highly reliable test would be necessary to achieve a sufficient reliability for the decision to pass or fail. This is not surprising to the extent that on a test aiming for the usual measurement reliabilities, a large portion of the questions display good discriminatory properties for the majority of the examinees, but give little information regarding the separation of the sparsely populated extreme groups. One approach – albeit difficult to implement at universities – would be the administration of two tests: the first serving the usual assessment of student performance and the second specifically for identifying masters and non-masters with questions specifically selected for this purpose (Kane [12], p. 430 has already suggested the latter). For the first test, a relatively high passing score is set, with which the probability of a non-master passing remains very low. The remaining group then consists of (poor) masters and non-masters who can be separated as well as possible by the second test. Methods for optimal question selection can be found in the literature using “(computerized) classification tests” (CCT) (e.g. [20], [15]).

## 6. Conclusion and outlook

The method of Douglas and Mislevy is suitable for analyzing the decision accuracy and consistency of overall decisions concerning assessments composed of multiple parts and for which the overall pass/fail decision is the result of a complex combination of individual scores. Above all, the conjunctive combinations (each individual test must be passed) and disjunctive combinations (only one of multiple tests must be passed; this applies for repeated tests) are of practical importance.

The graded course credit for a cluster of subjects (*fächerübergreifender Leistungsnachweis*) was selected as being exemplary of German medical education at present. In this testing situation, theoretical and practical assessments in different subjects are combined and, in order to pass overall, all of the components must be passed. Students have the possibility to repeat each individual test twice.

Using the method of Douglas and Mislevy, the decision accuracy and consistency for giving the graded course credit could be successfully analyzed; there was a high degree of congruence between the model and the data.

The analysis also revealed a significant issue concerning tests and low failure rates: these can only be reliably identified with difficulty for tests that comply with the usual demands for a sufficient reliability. Identifying masters and non-masters would require targeted classification tests with an appropriate selection of questions.

An analysis of the decision accuracy and consistency should generally be carried out on the relevant tests. The limitation of using the normal distribution model still needs to be viewed as a substantially limiting factor; it is to be hoped that suitable methods with weaker distribution assumptions (e.g. multivariate beta-binomial distributions) or distribution-free methods are developed.

## Competing interests

The authors declare that they have no competing interests.

## Figures and Tables

**Table 1:**
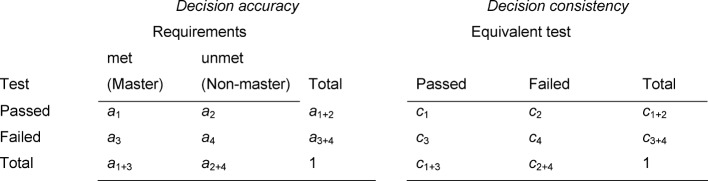
Contingency tables for decision accuracy and decision consistency. The a_i_ values represent the relative proportions of the scores on a test depending on whether students who fulfill the minimum requirements pass or fail (left). For example the value for a_2_ indicates the percentage of students who do not have sufficient knowledge/skills (non-master), but despite this have passed. In the case of two fully equivalent tests, c_i_ gives the analogous values. As a result of the equivalence of both tests c_2_=c_3_.

**Table 2:**
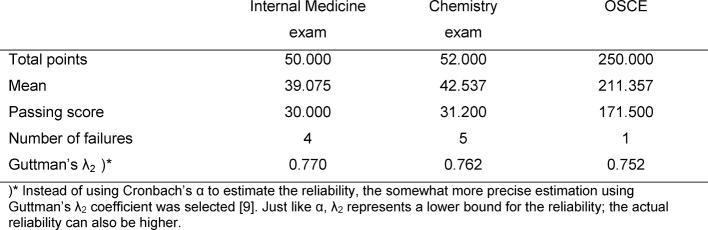
Basic data from the tests for graded credit in Internal Medicine/General Medicine/Clinical Chemistry during the winter semester 2012-13 (only examinees who took all three components: *N*=147).

**Table 3:**
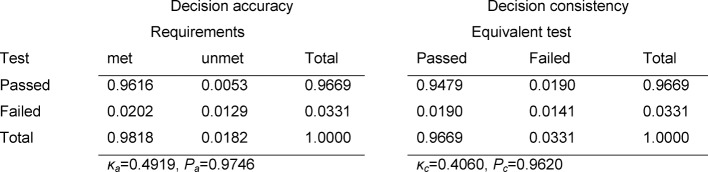
Decision accuracy and consistency for the exam in Internal Medicine

**Table 4:**
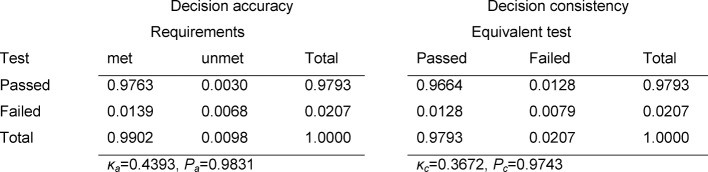
Decision accuracy and consistency for the exam in Clinical Chemistry

**Table 5:**
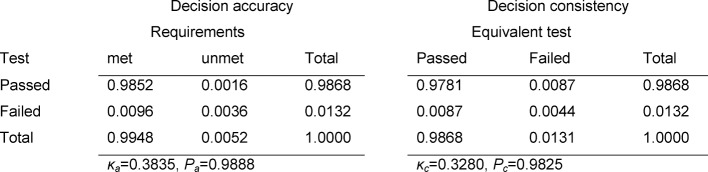
Decision accuracy and consistency for the OSCE in Internal Medicine

**Table 6:**
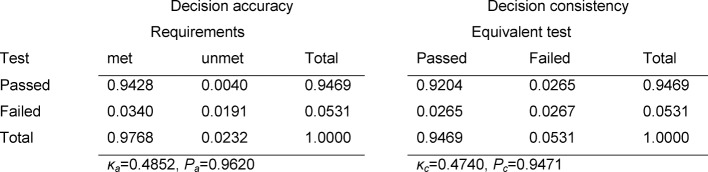
Decision accuracy and consistency for graded credit in the subject cluster Internal Medicine/General Medicine/Clinical Chemistry (conjunctive combination)

**Table 7:**
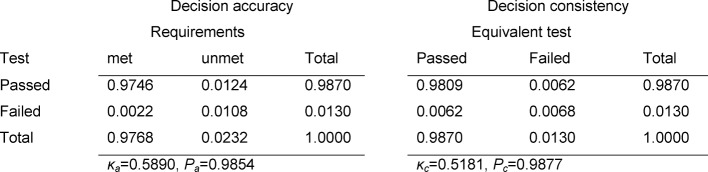
Decision accuracy and consistency for graded credit in the subject cluster Internal Medicine/General Medicine/Clinical Chemistry with the chance to repeat each test twice (see Figure 2)
